# Achieving vaccination justice: A call for global cooperation

**DOI:** 10.1371/journal.pgph.0000036

**Published:** 2021-10-13

**Authors:** Ellen Johnson Sirleaf, Helen Clark

**Affiliations:** 1 Presidential Center for Women and Development, Monrovia, Liberia; 2 Patron, The Helen Clark Foundation, Auckland, New Zealand; PLOS Global Public Health, UNITED STATES

The COVID-19 pandemic has exposed the reality–and the fragility–of our global interdependence like never before. The Independent Panel for Pandemic Preparedness and Response, which we were honoured to chair, looked frankly and fearlessly at the international response to COVID-19 up until the time we reported to the World Health Assembly in May 2021 [1]. We heard testimony from communities and those at the frontlines of the response who were unstinting in their efforts, often at great personal cost. These continuing challenges are not an inevitable force of nature. The evidence we gathered showed a series of systemic failures at every step in the path, from alert to early and then sustained response.

Core to the Panel’s diagnosis was that the root cause of these failures was a lack of decisive leadership directed towards cooperation and solidarity. The pandemic was worsened by pitting the economy against public health and the gross failure to protect the most vulnerable and give them the means to avoid exposure to the virus. Global health security metrics failed to predict which nations would be best prepared for the pandemic: they presumed that disease would emerge from poverty and the problem was a lack of capacity. Instead, it was a failure of politics–delay, neglect, and ‘me first’ responses.

The pandemic has caused the worst combined health and economic crisis for a century, creating the worst peacetime recession since the Great Depression [2], affecting half the world’s school population causing 100 million additional children to fall below minimum reading proficiency levels [3], and sending some 120 million additional people into extreme poverty [4], setting back progress for decades.

Unless fundamental change is made to the international system, then at any moment we risk another outbreak turning into another pandemic. The Panel recommended a comprehensive overhaul: making the alert system fit-for-purpose including accelerating the processes governed by the WHO International Health Regulations and matching them to the speed of agile, real-time surveillance building on initiatives like the Epidemic Intelligence from Open Sources collaboration; giving WHO the authority, independence and financing it needs to do the job the world expects of it; agreeing a framework convention to give the backing of binding international commitments to pandemic preparedness and response; creating a standing platform to accelerate response measures; ensuring countries build resilience; and supporting countries to do better through inclusive and transparent peer review of whole-of-society preparedness.

Bringing the recommendations together was our call for the elevation of pandemic risk to the highest levels of national and global leadership and attention. Pandemics pose the existential risk of system collapse. This risk goes beyond a single sector and a single nation. It demands permanent vigilance and the capacity to escalate response to the full degree necessary when an epidemic emerges.

A global body is needed to hold the system to account. Not to hold ‘others’ to account, but to hold ourselves to account. It needs to represent the apex of global leadership–and so it must be at the level of Heads of State and Government together with the best minds and most compelling moral voices the globe can provide. It must not fragment global decision-making, but bring it together. It needs to exercise legitimate and inclusive authority–for that reason we recommended that two of its chairs be nominated by the United Nations General Assembly and one by the G20. If the global community is serious about committing to ‘never again’ then a United Nations General Assembly Special Session is needed to bring the world together around a negotiated political declaration which charts the course for a safer future.

Financing is one of the key levers for accountability and that is why we recommend the Global Health Threats Council’s functions include oversight of a new financial mechanism, marrying incentives for investment in preparedness with a pool of funding available at short notice to meet pandemic response needs with the urgency they require. Improving global governance without finance will lack leverage, but improving financing without the right governance oversight will lack strategy.

Since our report there have been signs of global convergence around the key principles of inclusive, fundamental reform. United Nations Secretary-General António Guterres has asserted our recommendations “must be a starting point for urgent reforms to strengthen the global health architecture” [5]. The need for a leader-level global health threats council has been widely embraced, including by the G7 [6], the G20’s high level panel for financing the global commons for pandemic preparedness and response [7] and US President Biden the target set for the global COVID-19 summit he convened in September 2021 [8].

But despite the convergence around principles, this agreement is worth little if it is not accompanied by action in practice.

Mindful of the still-raging pandemic, in May 2021 we recommended urgent and immediate actions, including the need for high-income countries with an adequate vaccine pipeline to redistribute a billion doses to low- and middle-income countries by 1 September 2021. When the deadline came, barely a tenth of the target had been met. At 22 September 2021 only 3.3% of the population of low-income countries had received at least one vaccine dose, while in high-income countries the rate was 61.5% [9, 10].

Every week that goes by with a widening vaccination gap between rich and poor corrodes global trust. Abandoning countries to face the pandemic threat without the defences they need is both cruel and senseless.

Achieving vaccination justice is the first great test of this pandemic era–it requires targets and aspirations for vaccine access to be determined by health criteria not a country’s economic status, and timely delivery not a two-speed world where high-income populations are fully immunized within months but the poor are denied access for years. Meeting the vaccine justice test will signal that we have both understood the interdependence that determines our planetary future and have the capacity to act on it. Failing the test will condemn us to a ‘forever crisis’ of insecurity and recrimination as virus variants are given free rein and new vaccines struggle to keep up.

The COVID-19 pandemic demonstrates the peril of leaving inequity to fester, whether national impoverishment, gender and racial inequality, or chronic non-communicable disease. To end this pandemic and prevent the next one, we have to face up honestly to the mis-steps that were made, and be prepared to repair the global system fundamentally. Just as successive waves of COVID-19 demonstrate that half-measures do not work against this virus, incremental reform designed to leave vested interests in place will not create the security and protection we need.

We are at the point where we can make the turn towards a more equitable, safer, and sustainable world. Now is no time for differences in perspective to distract from that goal. We cannot afford to miss this turn.
